# Evaluation of serum cyfra21 in patients with pleural effusion

**Published:** 2012-10-30

**Authors:** Q Azimi, B Rezadoost, M Jalali Nadoushan, A Davati

**Affiliations:** 1 Dept. of Internal Medicine, Shahed University, Tehran, Iran; 2 Dept. of Pathology, Faculty of Medicine, Shahed University, Tehran, Iran; 3 Dept of Social Medicine, Shahed University, Tehran, Iran

**Keywords:** Cyfra21-1, pleural effusion, malignancy

## Abstract

**Background:**

The aim of this study is to determine the level of serum cytokeratin19 fragment (cyfra21-1) in patients with benign or malignant pleural effusion and evaluation the sensitivity and specificity of this tumor marker.

**Method & Material:**

We prospectively evaluated 98 patients (39 malignant, 54 benign and the results were inconclusive in 5) with pleural effusion. The diagnosis of malignant pleural effusion was defined by cytological or histological results. We used serum cyfra21-1 criteria from literature of Roch company. Level of the marker was determined using electrochemiluminescence.

**Results:**

There was statistically significant difference between mean of serum cyfra21-1 levels of benign (3.9±7.6) and malignant(19.5± 30) pleural effusion (p=0.01). In addition the sensitivity, specificity, positive predictive value and negative predictive value of serum cyfra21-1 at the cut off point of 3.3 ng/ml , was 84% , 61%, 61% and 84 % respectively.

**Conclusion:**

Serum cyfra21-1 is useful as a measure in differentiating malignant from benign pleural effusion.

## Introduction

The fluid accumulation in the pleural area is called pleural effusion. Any factor that causes changes in hydrostatic or oncotic pressure of parietal pleural capillary vessels or changes the permeability rate of pleural membrane can cause pleural effusion.(1) Most important causes of pleural effusion are pneumonia (the most common cause), lung carcinomas, metastatic pleural disease, gastrointestinal diseases, tuberculosis, rheumatologic diseases, post-cardiac injury syndrome, uremia, drug reactions, heart failure, asbestosis and hypoalbuminemia after burning.(1) Nowadays non-invasive methods such as chest radiography, ultrasonography, laboratoty tests (measurement of electrolytes, blood cell count & diff, blood urea nitrogen, creatinine, lactate dehydrogenize) and also invasive methods such as thoracoscopy, thoracocentesis, cytology and pleural biopsy is used to diagnose the cause of pleural effusion. Indeed each course has its own problems.(2) It seems that noninvasive tests are necessary, but according to the overlap of test results in various diseases and spending time and cost, lowest diagnostic results are achieved.(2) Among invasive tests, thracoscopy increases risks such as pneumothorax, hemothorax and implanted tumors in another places(4%)(3), traceocentesis and cytology tests are successful and diagnostic in 50 to 70 percent(4) and the diagnostic value of pleural biopsy is only almost 7 percent more than invasive methods.(5) In addition, these methods are very painful and expensive, so using more specific and reliable methods, according to the patient's history, seems necessary. One of these methods is using serum tumor markers, although it is not too much specific at early stages of tumors, it is a reliable method in the end stages of cancers and can be helpful diagnosing benign cases from malignancies.(5) These tumor markers include Neurocarcinoma specific enolase (NSE), Carcinoembryonic antigen (CEA), Cyfra21 (fragmented cytokeratin19), Cancer Antigen 19-9 (CA19-9), Cancer Antigen 15-3 (CA15-3), granulocyte colony stimulating factors (G-CSF).(2) Cyfra21 is a chopped cytokeratin19, which is soluted in serum and it is a useful tumor marker in blood circulation. In some studies it was shown that using this tumor marker is useful in diagnosing metastatic lung tumors and end-stage lung cancers and helps differentiating benign diseases inducing pleural effusions from malignant diseases.(6) Of course, different sensitivity and specificity of this tumor marker was reported in research results. The study of Lee JH et al in 2005 showed that levels of cyfra21 in patients with cancer are higher than tuberculosis.(4) Procel JM et al studied several tumor markers including cyfra21 in 2004. They reported that the sensitivity of this tumor marker, alone, is 22% and with other studied tumor markers (CEA, CA125, and CA15-3) is 54%.(7)

We have researched on serum levels of Cyfra21in patients with pleural effusion and have studied its relationship with malignancy (existence of malignant cells based on pathological findings).

## Materials and Methods

This study is a cross sectional study. It was done on all patients (97 patients) with pleural effusion who were admitted in Tehran's Mostafa Khomeini hospital during 18 months (2008-2010). After initial approval, the data form was completed. Serum samples were taken routinely from all hospitalized patients. The serum samples were storaged at -70 ° C temperature. The levels of cyfra21 in these samples were measured by using Electro chemiluminescence method (ECL) (Roche, Germany). The patients with pleural effusion were separated into groups of benign and malignant types of diseases causing pleural effusion and pulmonary and non-pulmonary causes. The results were entered to the data-based form and finally they were analyzed by SPSS vrsion 16, with Mann-Whitney test and ROC plot.

We used clinical findings and paraclinical methods such as chest radiography, ultrasonography, laboratory and invasive methods such as thracoscopy, thoraceocntesis, pleural cytology and lung biopsy to differentiate benign diseases from malignant diseases. To determine the sensitivity and specificity of this tumor marker for diagnosing malignant pleural effusion, the cutoff point of the kit was 3.3ng/ml. This information was released by the company who produced the kit.

## Results

The study was done on 98 patients (64 males and 34 females). The range of the patient's age was 40 to 100 years. All the patients had pleural effusion. Among them 39 cases had malignancy and 54 cases had benign tumors. 5 case were not diagnosed. The cyfra21 level in the undiagnosed patients was 0.87, 1.35,1.6, 1.95,and 2.37.

The average of levels of cyfra21 in the group with malignant disease was showed in table 1 (p < 0.001). The cyfra21 level in male and female patients and in pulmonary and non pulmonary patients is also showed in table 1.

**Table 1:** The mean of cyfra21 pleura effusion, both sexes, and pulmonary and non- pulmonary diseases

	Mean±SD
**Causes of pleural effusion**	
Malignant	19.5±30*
Non malignant	3.9±7.6
**Gender**	
Male	12.1±27
female	8.9±13
**Source of diseases**	
Lung diseases	11.18±28**
Other diseases	9.9±14.2

**Figure 1:**
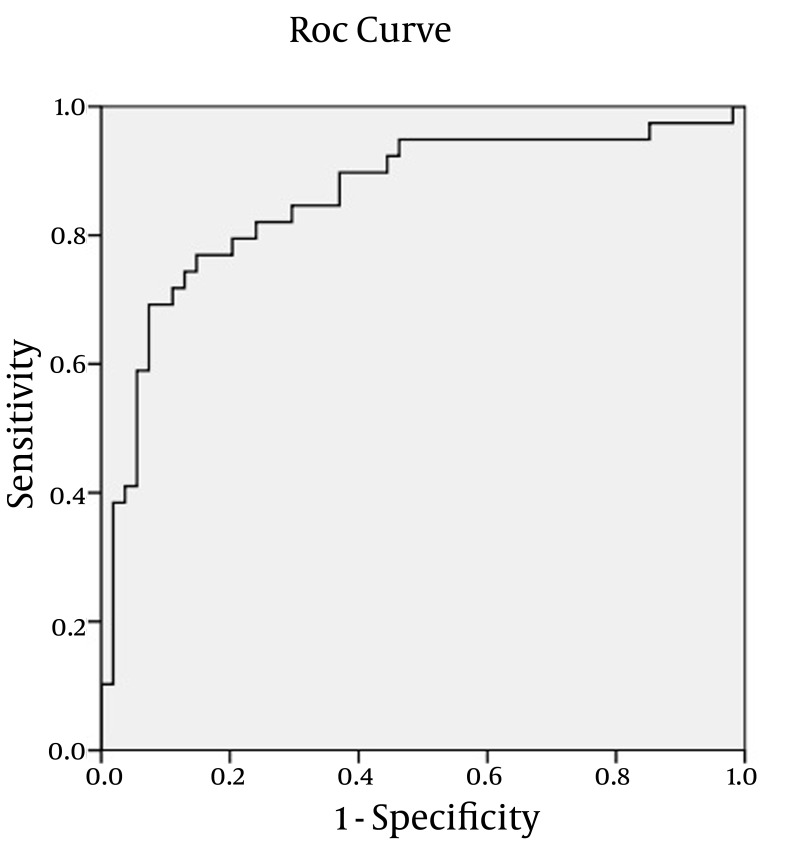
Receiver operating characteristic (ROC) curves. ROC curves show the relationship between sensitivity and specificity for Cyfra-21

For providing a pure index of accuracy, we showed accuracy by ROC plot demonstration (Figure 1). Area under plot is 0.858 with p= 0.001.

The sensitivity of cyfra21 as a tumor marker was 84%, the specificity was 61%, positive predictive value (ppv) was 61% and negative predictive value was 84%.

## Discussion

The results showed that cyfra21 blood levels in malignant cases were higher than in non-malignant cases and there was a significant association between blood levels of this tumor marker with malignancy.

Several studies have been done on this tumor marker and its relationship with malignant pleural effusions in the world, which had different results. Wanger IC and his colleagues studied on 85 cases with pleural effusion in 2007 in Brazil and showed that cyfra21 is helpful in diagnosing benign cases from malignant cases and its sensitivity was almost 71.4% (8). Their results were similar to ours, which can be because the In vitro methods (ECLIA) and the cutoff point of the two studies were the same.

Another research was done by Procel JM et al in 2004 in Spain. They studied on 416 patients with pleural effusion and several tumor markers including cyfra21. They reported the sensitivity of cyfra21 alone just 22% and 54% with other tumor markers such as CEA, CA125, CA15-3.7 Although the reported number of sensitivity for cyfra21 of this study differs from ours, higher amount of samples and analyzing three more tumor marker in their study makes their research more valuable than ours.

Also Dejsomritrutai W et al studied the diagnostic importance of cyfra21 on 62 patients with pleural effusion (27 malignant and 35 benign) in 2001 in Thailand. They showed that cyfra21 has high sensitivity (81.5%) and specifity (97.1%) to differentiate malignant and benign cases.(6) Although the research's results confirms our results about the efficacy of cyfra21 in differentiating benign from malignant cases, it's number of specifity differs a lot that could be due to the differences in experimental methods (ELISA) and the cutoff point which is defined for distinguishing malignant cases (2.5ng/ml). Our sample size was more and we used a more reliable cut off point (3.3ng/ml) than their study. According to these, it seems that our specifity is more valuable and reliable than their study.

Lee and colleagues also studied several tumor markers including cyfra21-1 on 50 patients whom 34 cases had lung cancer and 16 cases had tuberculosis. They reported that serum cyfra21-1 had 45% sensitivity and 100% specificity for diagnosing cancer. They used radioimunoassay (RIA) method for measuring the level of cyfra21-1 and used 3.3 ng / ml as their cutoff point. Differences in experimental method and type of sampling can be the reason of the differences between their results and ours. They also showed that cyfra21-1 is particularly very helpful in diagnosing lung SCC and its serum levels in this tumors is much more higher than other lung cancers such as adenocarcinoma and small cell carcinoma. He also claimed that the simultaneous measurement of pleural fluid cyfra21-1 could increase the diagnostic sensitivity up to 70%.(4)

According to the results we can say that although cyfra21 is a useful tumor marker for distinguishing benign cases from malignant cases , its sensitivity is not enough to exclude or confirm malignancy. Using more sample size, measuring other related tumor markers and measurement of pleural fluid levels of cyfra21 simultaneously could increase the reliability of the research.
